# Phytohormone abscisic acid elicits positive effects on harmaline‐induced cognitive and motor disturbances in a rat model of essential tremor

**DOI:** 10.1002/brb3.2564

**Published:** 2022-04-05

**Authors:** Mohammad Shabani, Reyhaneh Naderi

**Affiliations:** ^1^ Neuroscience Research Center, Neuropharmacology Institute Kerman University of Medical Sciences Kerman Iran

**Keywords:** abscisic acid, cognitive impairments, harmaline, motor impairments, tremor

## Abstract

**Objective:**

Essential tremor (ET) as a neurological disorder is accompanied by cognitive and motor disturbances. Despite the high incidence of ET, the drug treatment of ET remains unsatisfactory. Recently, abscisic acid (ABA) has been reported to have positive neurophysiological effects in mammals. Here, the effects of ABA on harmaline‐induced motor and cognitive impairments were investigated in rats.

**Methods:**

Male Wistar rats weighing 120–140 g were divided into control, harmaline (30 mg/kg, ip), ABA vehicle (DMSO+normal saline), and ABA (10 μg/rat, icv, 30 min before harmaline injection) groups. Exploratory, balance and motor performance, anxiety, and cognitive function were assessed using footprint, open field, wire grip, rotarod, and shuttle box tests.

**Results:**

The results indicated that ABA (10 μg/rat) can improve harmaline‐induced tremor in rats. The administration of ABA significantly increased time spent on wire grip and rotarod. In addition, ABA had a promising effect against the cognitive impairments induced by harmaline.

**Conclusion:**

Taken together, ABA has positive effects on locomotor and cognitive impairments induced by tremor. However, further studies are required to determine the exact mechanisms of ABA on the ET.

## Graphical abstract

Abscisic acid affected explorative and gait disturbances induced by harmaline. Abscisic acid typically ameliorated harmaline induced tremor. Pretreatment with Abscisic acid protects against harmaline‐induced alterations.

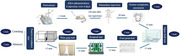

## 1 INTRODUCTION

Essential tremor (ET) is a prevalent movement disorder in adults, which has destructive effects on the quality of the patient's life. This neurodegenerative disorder is characterized by distal postural and kinetic tremor mainly involving the upper limbs (Louis, 2005). It has been demonstrated that the risk of developing dementia and cognitive impairments is elevated in patients with ET (Deuschl & Elble, 2009; Janicki et al., 2013). Although exact mechanisms of inducing tremor have not been completely clarified, it seems that hyperactivity of the excitotoxic neurons is the cause of ET (Choe et al., 2016; Shaikh et al., 2008).

Recent studies confirmed this hypothesis using harmaline‐induced tremor in animals (Abbassian, Esmaeili, et al., 2016; Abbassian, Whalley, et al., 2016 ; Vaziri et al., 2015). Harmaline, an alkaloid metabolite of the plant *Peganum harmala* has been demonstrated to be a tremorigenic agent (Park et al., 2015). In addition, it has been shown that harmaline disrupts cognitive functions in rodents (Du & Harvey, 1997). The administration of harmaline induces tremor with oscillation frequency in between 10 and 12 Hz in rats (Miwa, 2013). Harmaline increases firing rate of the neurons arising from the inferior olive nucleus (ION) and ending on Purkinje cells (PCs) of the cerebellar cortex (Handforth, 2012), which leads to an enhancement of glutamate release in the cerebellum (Beitz & Saxon, 2004; Gołembiowska et al., 2013) and potentiation of complex spike discharges of PCs (Lamarre et al., 1971). Therefore, it seems compounds with reducing potential of the neuronal excitability can relieve ET symptoms (Arjmand et al., 2015).

The isoprenoid abscisic acid (ABA) is known as a plant hormone that regulates fundamental physiological functions including plant growth and development, seed dormancy and germination, senescence, and plant responses to stresses (Cutler et al., 2010; Finkelstein, 2013). Recent studies have indicated that this substance also exists in various tissues and cells of animals, particularly, in mammalian brain (Le Page‐Degivry et al., 1986; Qi, Ge, et al., 2015; Qi, Zhang, et al., 2015). Abscisic acid is implicated in several physiological functions including immune processes (Bruzzone et al., 2007; Magnone et al., 2012), stem cell expansion (Scarfì et al., 2008), and glucose homeostasis (Bruzzone et al., 2008, 2012; Guri et al., 2007), ABA is also found as an anti‐inflammatory (Guri et al., 2007, 2008, 2011), anti‐oxidative (Soti et al., 2019), anti‐apoptotic (Rafiepour et al., 2019), anti‐atherosclerosis (Guri et al., 2008), and anti‐cancer (Ma et al., 2006) factor in animals.

It has been reported that ABA can be obtained through diet (Magnone et al., 2015) and also produced in the various tissues of the mammal's body, especially brain (Le Page‐Degivry et al., 1986; Qi, Ge, et al., 2015). Some studies have demonstrated that ABA has neuroprotective effects in animals. Both systemic and central administration of ABA has positive effects on spatial learning and memory performance and mood state of rats (Naderi et al., 2017; Qi, Ge, et al., 2015). In addition, ABA is able to improve cognitive impairments induced by Alzheimer's (Khorasani et al., 2019) and diabetes (Kooshki et al., 2020) diseases in rats. It has been indicated that ABA restores neuroinflammation, neurogenesis, and cognitive deficits in a rat model of high‐fat diet (Ribes‐Navarro et al., 2019; Sánchez‐Sarasúa et al., 2016).

Previous studies have shown that ABA is produced in the brain (Le Page‐Degivry et al., 1986; Qi, Ge, et al., 2015; Qi, Zhang, et al., 2015) and plays a neuroprotective role in rats (Khorasani et al., 2019; Naderi et al., 2017; Qi, Ge, et al., 2015). However, ABA's potential to improve cognitive and motor performance in rats with harmaline‐induced tremor has not yet been determined. Therefore, this study was designed to evaluate the effect of central administration of ABA on harmaline‐induced cognitive and motor disturbances in a rat model of ET.

## 2 MATERIALS AND METHODS

### 2.1 Animals

Male Wistar rats aged 5−6 weeks weighing 120−140 g were used in this study. Animals were housed in a room with controlled photo period (12‐h light/dark cycle) with access to food and water ad libitum. All procedures were carried out according to animal ethics committee (Ethics code: KNRC/00/002) guidelines of the Kerman Medical University.

### 2.2 Drugs and experimental design

Harmaline hydrochloride dihydrate and (±)‐cis, trans‐ABA were purchased from Sigma‐Aldrich (USA). Harmaline dose was selected based on previous studies in our laboratory and 30 mg/kg is considered as an appropriate dose to show signs of tremor (Abbassian, Whalley, et al., 2016). Pilot studies (*n* = 16, Dosages: 10, 30,60, 120; 4 rats/dose) revealed that 30 mg/kg harmaline induced stable tremor in this population for the duration of the testing period (180 min).

**FIGURE 1:**
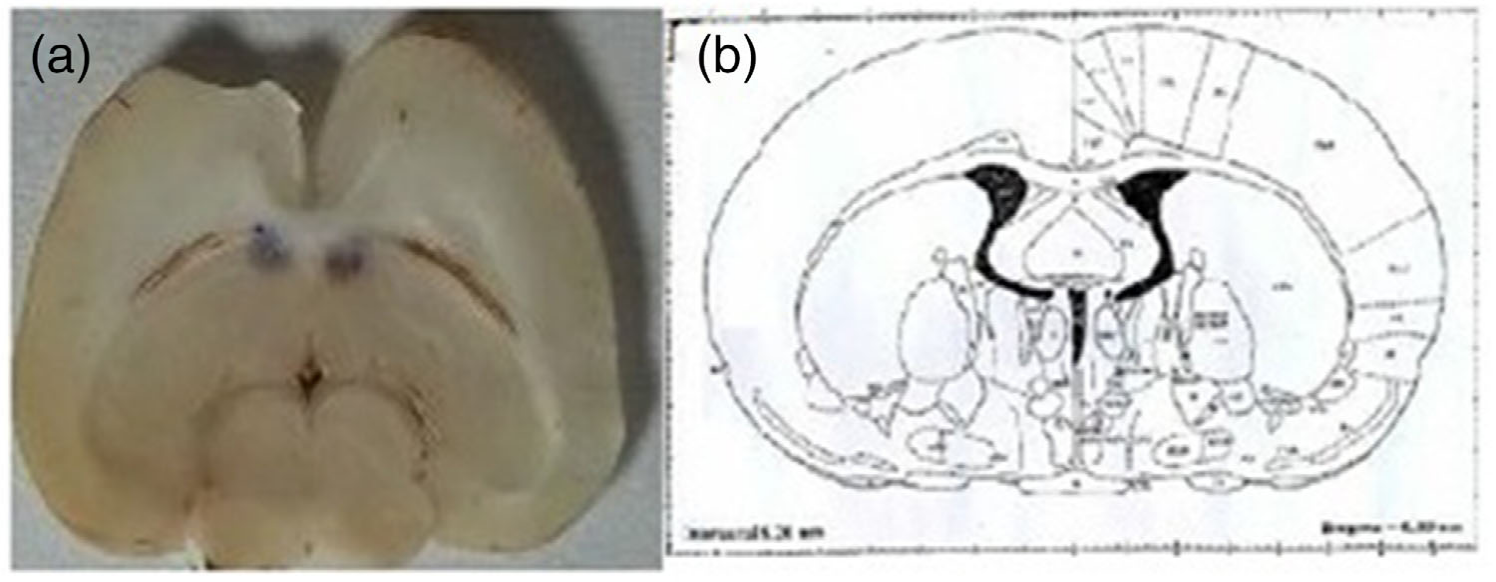
A: a typical coronal section displays unilateral injection site in the lateral ventricles, B: a schematic coronal section of lateral ventricles reproduced from the atlas of Paxinos and Watson

**FIGURE 2:**
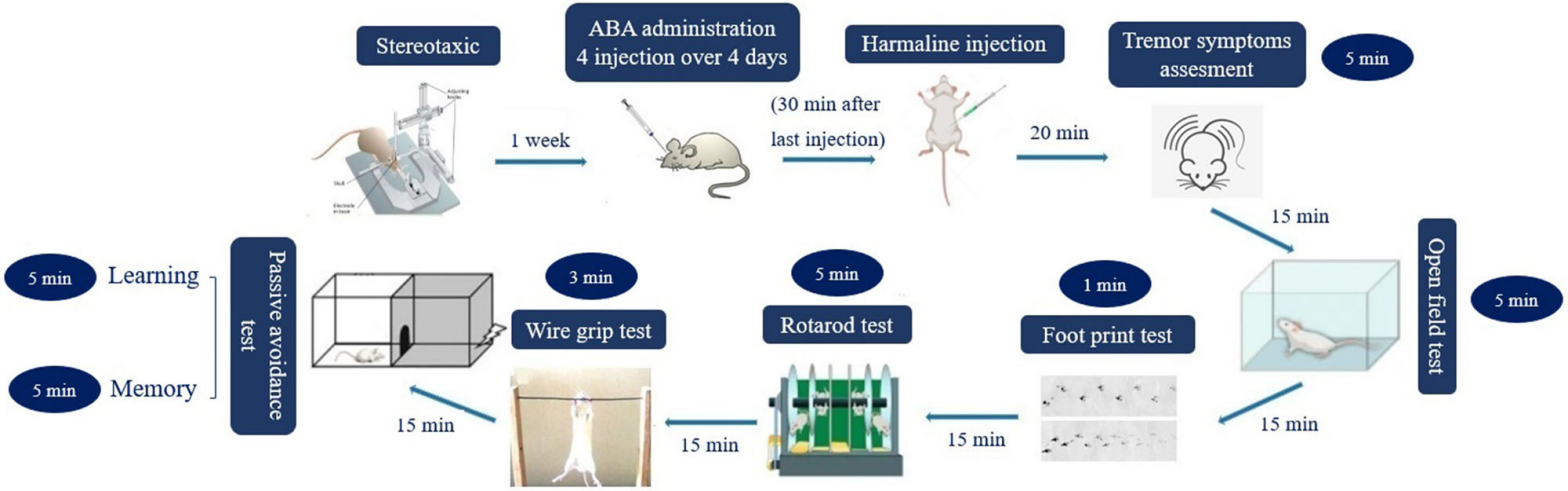
The experimental design and study timeline

Abscisic acid was dissolved in the sterile saline solution (0.9% w/v sodium chloride) with dimethyl sulfoxide (DMSO) in a ratio of 2:1 (v/v) and administered intracerebroventricularly (icv) through a 27‐gauge internal cannula connected via polyethylene tubing to a 1 μl Hamilton syringe. The drug's spreading zone in the lateral ventricles is shown in Figure 1. Harmaline was dissolved in normal saline and injected intraperitoneally (ip) 30 min after ABA administration. The animals were divided into control, saline (saline+30 mg/kg harmaline, ip), sham (ABA vehicle, icv, 30 min before harmaline injection), and harmaline + ABA (10 μg/rat, icv, 30 min before harmaline injection) groups (*n *= 7). The experiments were performed 20 min after harmaline injection with suitable interval among each test in the following order: Observation, open field test, rotarod, wire grip test, and footprint (Abbassian, Esmaeili, et al., 2016). A brief experimental design timeline is depicted in Figure 2. Each group went through five different behavioral studies, which were performed 30 min after harmaline injection with sequentially 15 min rest intervals among each assay in the following order: Tremor score assessment (5 min), open field test (5 min), footprint (1 min), rotarod (5 min), wire grip (3 min), and passive avoidance task (learning phase: 5 min; memory phase 24 h after the learning phase: 5 min).

### 2.3 Surgery

Rats were anesthetized with a mixture of ketamine (50 mg/kg, ip) and xylazine (5 mg/kg, ip) and placed in a stereotaxic apparatus (Stoelting, USA). Guide cannulas were implanted bilaterally into right and left ventricles according to the atlas of Paxinos and Watson. Stereotaxic coordinates of ventricles were AP = 1.6 mm from Bregma, ml = ± 0.8 mm from the midline, and DV = 3.4 mm from the skull surface. The cannulas were fixed to the skull using two screws and dental cement and then closed with a stylet. After the surgery, the animals were kept in individual cages and given recovery period a week before experiments.

### 2.4 Tremor

Tremor was rated by an observer who was blinded to the treatment protocol. Twenty minutes after harmaline administration, the data were acquired during the open field test. Balance disturbances were scored as the following scale: 0: No tremor, 1: occasional tremor affecting only the head and neck, 2: intermittent (occasional tremor affecting all body parts), 3: persistent (tremor affecting all body parts), 4: severe (persistent tremor rendering the animal unable to stand and/or walk) (Al Deeb et al., 2002).

### 2.5 Open field test

The apparatus consisted of a square arena (90 × 90 × 45 [H] cm) made of Plexiglas, which was divided into central and peripheral regions. Each rat was placed in the center of the arena and the behavioral parameters were recorded for 5 min and assayed with offline analysis (Ethovision7.1, Noldus Information Technology, The Netherlands). Total distance moved (TDM, cm) velocity, mobility, and time spent in the center were recorded as the locomotor activity and anxiety like behavior, respectively. The maze was cleaned with 70% ethanol and dried between sessions (Nazeri et al., 2014).

### 2.6 Footprint

Footprint test was used to assess the walking patterns and gait kinematics. Hind paws of the animals were marked with nontoxic ink. The rats were allowed in a clear Plexiglas tunnel (100 cm [L] × 10 cm [H] × 10 cm[W]), ending in a darkened cage. A sheet of white absorbent paper (100 cm × 10 cm) was placed on the floor of the tunnel. The resulting tracks provide the spatial relationship of consecutive footfalls from which rat stride length and width were measured.

### 2.7 Rotarod test

Motor coordination and balance were assessed by accelerating rotarod apparatus (Hugo Sachs Electronik, Germany). All rats were trained on the rotarod (8 rpm, 5 min) 24 h before the test. The rotarod experiment started at a speed of 10 rpm to the maximum speed of 60 rpm within 5 min. The protocol of the rotarod test included three trials with 20‐min intervals. The average of staying time on the rod was calculated for each rat during the three trials (Shojaei et al., 2012).

### 2.8 Wire grip test

The muscle strength of the animals was evaluated using wire grip test. During the test, each rat was suspended on a horizontal steel wire. Each rat was suspended by both forepaws from a horizontal steel wire (80 cm long, 7 mm diameter), which was suspended 45 cm from the ground. The animals’ forepaws were put in contact with the steel wire and released whenever they grasped the wire. The animals underwent three trials with 20 min rest interval, and the falling latency was recorded using a stopwatch for each rat.

### 2.9 Passive avoidance test

Before the test, rats were placed individually in the light chamber of the apparatus. Ten seconds later, the door was opened and the animal was allowed to go to the dark chamber without electric shock for 30 s. Then, the door was closed and the animal was returned to the home cage. One hour later, the learning phase was performed and each rat was placed into the light chamber. Once the animal entered the dark compartment, the door was closed and an electrical stimulation (0.5 mA, 50 Hz) was delivered to the animal's feet through the stainless steel rods for 2 s. This step was repeated at 30‐min intervals until the animal learned to avoid the dark chamber, and the number of shocks for learning was recorded. Memory retrieval was examined 24 h after the learning phase. The animal was placed in the light chamber (door closed) and 10 s later, the door was opened. The latency to enter the dark chamber (step‐through latency; STL) was recorded in 300 s (Razavinasab et al., 2016).

### 2.10 Statistical analysis

Statistical analyses were performed using SPSS Statistics version 16. The collected data were analyzed using one‐way ANOVA and expressed as mean ± SEM. Post‐hoc analyses were conducted using the Tukey's test. A *p* value of <0.05 was considered to be statistically significant.

## 3 RESULTS

### 3.1 Effect of ABA on tremor score and gait disturbance in harmaline‐treated rats

**FIGURE 3:**
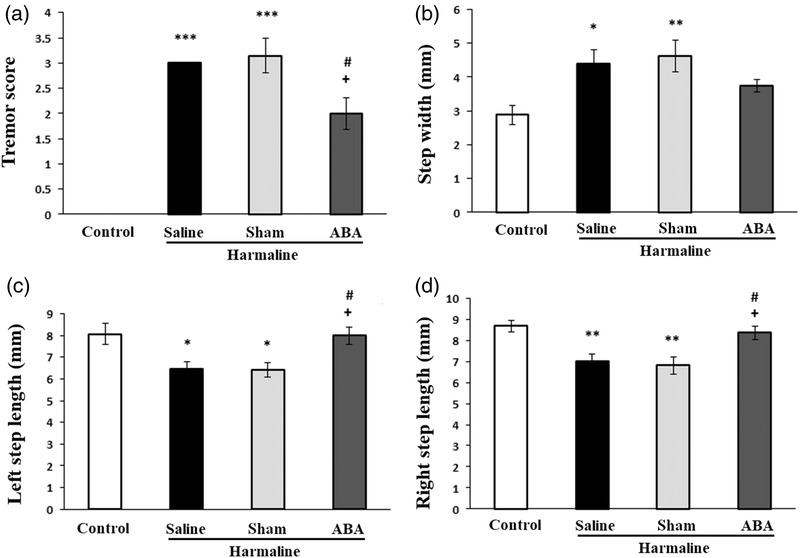
Effect of ABA on tremor scores (A), step width (B), left (C) and right step length (D) after harmaline administration. Data presented as mean ± SEM. *p < 0.05, **p < 0.01 and ***p < 0.001 versus control group; ^+^ *p* < 0.05 versus harmaline group; ^#^ *p* < 0.05 versus sham group

Figure 3 shows that administration of harmaline induced a significant and persistent tremor in rats [*F*(3, 24) = 39.839, *p* = 0.001]. The score of tremor scale significantly increased in rats treated with harmaline as compared to the control group (*p* < 0.001). Microinjection of ABA could significantly attenuate the tremorgenic effects of harmaline as compared to control and sham animals (*p* < 0.05, Figure 3A).

As shown in Figure 3B, there were significant differences between experimental groups in step width [*F*(3, 24) = 4.705, *p* = 0.01]. Harmaline injection significantly increased step width as compared to the control group (*p* < 0.05). However, there was no significant difference between harmaline and ABA groups in step width demonstrating that ABA treatment was not able to ameliorate harmaline effects upon step width.

In addition, there were significant differences between experimental groups in left [*F*(3, 24) = 5.658, *p* = 0.004] and right [*F*(3, 24) = 7.377, *p* = 0.001] step lengths. The significant decreases in left and right step lengths were observed in the harmaline group as compared to the control group (*p* < 0.05) and (*p* < 0.01), respectively, (Figure 3C and D). Interestingly, ABA has been found to have adverse effects on left and right step lengths; hence, ABA significantly decreased left and right step lengths even from the level of harmaline group (*p* < 0.05, Figure 3C and D).

### 3.2 Effect of ABA on explorative and anxiety‐related behaviors in harmaline‐treated rats

**FIGURE 4:**
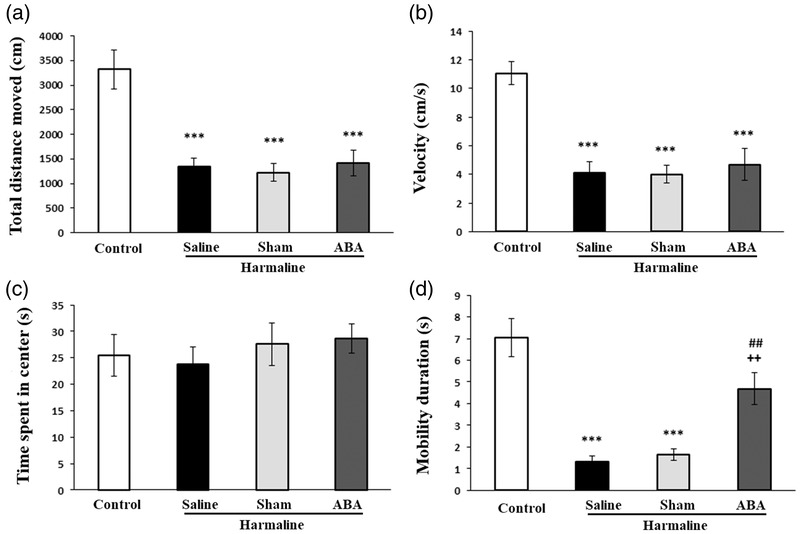
Effect of ABA on explorative and anxiety like behavioral changes induced by harmaline. Total distance moved (A), velocity (B) and rearing number time spent in center (C), and mobility duration (D). Data presented as mean ± SEM. ***p < 0.001 versus control group; ^++^ *p* < 0.01 versus harmaline group; ^##^ *p* < 0.01 versus sham group

Figure 4 shows there were significant differences between experimental groups in total distance moved [*F*(3, 24) = 14.023, *p* = 0.001], velocity [*F*(3, 24) = 16.243, *p* = 0.001], and mobility [*F*(3, 24) = 20.226, *p* = 0.001]. Total distance moved, velocity, and mobility were significantly decreased in harmaline‐treated rats as compared to the control group (*p* < 0.001). ABA administration could increase mobility in harmaline‐treated rats as compared to the harmaline and sham groups (*p* < 0.01, Figure 4D). However, microinjection of ABA had no significant effect on harmaline‐induced disturbances in total distance moved and velocity (Figure 4A and B). As shown in Figure 2C, there was no significant difference between groups in time spent in center.

### 3.3 Effect of ABA on balance and muscle strength in harmaline‐treated rats

**FIGURE 5:**
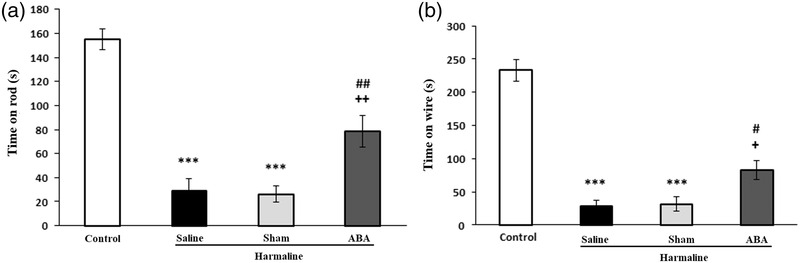
Effect of ABA on muscle strength and balance function after harmaline administration. Mean latency to fall in wire grip test (A), Mean of three repeated trials of the staying time on the rod (B). Data presented as mean ± SEM. ***p < 0.001 versus control group; ^+^ *p* < 0.05 and ^++^ *p* < 0.01 versus harmaline group; ^#^ *p* < 0.05, and ^##^ *p* < 0.01 versus sham group

The muscle strength of animals was evaluated by averaging time spent on the wire grip test in three sequential trials. As shown in Figure 5A, there were significant differences between experimental groups in the time period on the wire grip [*F*(3, 24) = 38.196, *p* = 0.001]. The rats of the harmaline group significantly spent shorter time period on the wire grip as compared to the control group (*p* < 0.001). Falling time significantly increased in ABA‐received rats compared to sham and harmaline groups (*p* < 0.01, Figure 5A).

As shown in Figure 5B, there were significant differences in balance function of rats in the rotarod test [*F*(3, 24) = 54.171, *p* = 0.001]. Harmaline‐received rats showed a significant reduction in the duration staying on rod during the three repeated trials (*p* < 0.001). However, pretreatment with ABA significantly increased mean time spent on the apparatus as compared to harmaline group (*p* < 0.05, Figure 5B).

### 3.4 Effect of ABA on learning and memory in harmaline‐treated rats

**FIGURE 6:**
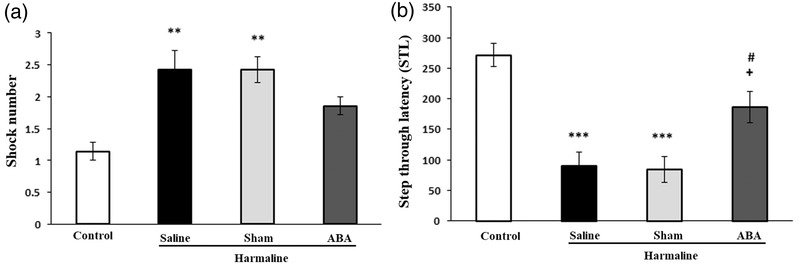
Effect of ABA on passive avoidance after harmaline administration. Number of shock (A), Step through latency (STL), (B). **p < 0.01 and ***p < 0.001 versus control group; ^+^ *p* < 0.05 versus harmaline group; ^#^ *p* < 0.05 versus sham group

Figure 6A shows there were significant differences between experimental groups in the number of received shocks [*F*(3, 24) = 8.760, *p* = 0.001]. Harmaline group had a higher number of received shocks as compared to the control group (*p* < 0.01), which implicates impaired learning. However, there was no significant difference in shock numbers between ABA‐received rats and the harmaline group (Figure 6A). In addition, there were significant differences between experimental groups in STL [*F*(3, 24) = 16.015, *p* = 0.001]. The results indicated that STL significantly decreased in harmaline group as compared to the control animals (*p* < 0.001). Microinjection of ABA prior to harmaline significantly inhibited the reducing effect of harmaline on STL (*p* < 0.05, Figure 6B).

## 4 DISCUSSION

In the present study, the possible protective effect of ABA was investigated on harmaline‐induced defects in balance, muscle strength, and cognitive function. The data showed that harmaline induced generalized tremor, which was manifested as several deficits including increased step width with simultaneously decreased step length bilaterally, balance disturbance, decreased muscle strength, and impairment of learning and memory in the passive‐avoidance test. Central administration of ABA prior to harmaline could attenuate tremorgenic effects of harmaline. In addition, ABA prevents harmaline‐induced defects in balance function and muscle strength of animals. The administration of ABA also reverses the destructive effect of harmaline on passive avoidance retention.

It has been demonstrated that administration of harmaline induces generalized transient tremor that lasts from minutes to hours (Miwa, 2013). Previous studies have been reported that harmaline impairs motion and cognitive functions in rats; the results of the present study confirm these defects caused by harmaline‐induced tremor (Abbassian, Esmaeili, et al., 2016; Aghaei et al., 2019; Janicki et al., 2013).

It is thought that harmaline exerts its excitatory effects on the nervous system by increasing the activity of the olivo‐cerebellar glutamatergic pathway. It has been indicated that AMPA and NMDA glutamate receptors are located in inferior olive and cerebellum (Chen et al., 2006; Paarmann et al., 2000; Petralia et al., 2004). Harmaline‐induced overactivity of climbing fiber of the inferior olive connected to PCs increases glutamate release in the cerebellum. Excessive glutamate release in the cerebellum leads to excitotoxic damage and degeneration in PCs (Beitz & Saxon, 2004; Gołembiowska et al., 2013; Lamarre et al., 1971). Glutamate receptor blockade through administration of the NMDA and AMPA antagonists attenuated harmaline‐induced hyperactivity (Du & Harvey, 1997; Eblen et al., 1996; Iseri et al., 2011; Paterson et al., 2009). Therefore, the glutamatergic system plays a fundamental role in the tremor induced by harmaline.

Abscisic acid is produced and released from cells in several areas of the brain including hypothalamus, hippocampus, cortex, and cerebellum (Qi, Ge, et al., 2015; Qi, Zhang, et al., 2015). Recent studies have shown that ABA has positive neurobiological effects in the nervous system of mammals. It has been indicated that the chronic systemic administration of ABA improves spatial learning and memory and induces exploratory activity (Qi, Ge, et al., 2015). We have also reported that the central injection of ABA has a positive effect on cognitive functions and elicits anti‐anxiety activity (Naderi et al., 2017). It has been demonstrated that treatment with ABA can change feeding behavior and body weight of rats (Soti et al., 2019). In addition, ABA has been shown to have anti‐atherosclerosis (Guri et al., 2010), anti‐depressant (Qi, Zhang, et al., 2015), anti‐nociceptive (Mollashahi et al., 2018), and anti‐cancer effects (Li et al., 2011). However, in the present study, the administration of harmaline or ABA did not influence anxiety‐like behavior. Since the open field is not a functional test to measure anxiety, the exact role of tremor and ABA in the modulation of anxiety‐like behaviors need to be evaluated through the elevated plus maze in further studies.

Previous studies have revealed that pain is associated with cognitive dysfunction (Kewman et al., 1991; McCarberg & Peppin, 2019). Painful conditions cause people to perform poorly in tests of intelligence, reasoning, and memory (Hart et al., 2000). In addition, it has been demonstrated that movement activity is changed in painful condition. Pain can produce a large range of movement changes from disturbance in motor activities to complete avoidance of painful movements and/or activities (Hodges, 2011; Merkle et al., 2020). Abscisic acid has been shown to have pain‐relieving property, which may be involved in the improvement of motor and cognitive impairments induced by tremor (Mollashahi et al., 2018).

It seems that the neuroprotective effects of ABA are mediated via its antioxidant capacity. ABA plays a fundamental role in the activation of antioxidant defense system and enhancement of antioxidant capacity in plants. It has been indicated that ABA induces the expression of antioxidant defense genes and increases activities of antioxidant enzymes in plants (Ming‐Yi & Jian‐Hua, 2004; Prasad et al., 1994; Sandhu et al., 2011). Furthermore, Soti and colleagues reported that chronic treatment of ABA increases the activity of antioxidant enzymes and antioxidant capacity in rats’ brain (Soti et al., 2019). Interestingly, ABA's antioxidant role in the improvement of 6‐OHDA‐induced cell damage has been demonstrated in a model of Parkinson's disease (PD) (Rafiepour et al., 2019).

Tao Hou et al. demonstrated that phaseic acid (PA), analogous of ABA, inhibits NMDA glutamate receptors during ischemic brain injury. They reported that PA blocks NMDA receptors in a noncompetitive manner similar to memantine, an uncompetitive antagonist of the NMDA receptors. Therefore, PA or its analogs can act as an endogenous and reversible inhibitor of glutamate receptors in mouse brain (Hou et al., 2016).

Both in vivo and in vitro studies indicated that ABA increases the expression and activity of PPARγ receptors. It has been shown that ABA exerts its anti‐inflammatory functions via PPAR γ‐dependent mechanism (Bassaganya‐Riera et al., 2010, 2011; Guri et al., 2011). Treatment with ABA inhibits diabetes‐induced learning and memory and synaptic plasticity destruction in rats through interaction with PPARγ receptors (Kooshki et al., 2020). The blockage of PPARγ receptors prevented ABA‐induced learning and memory improvement in streptozotocin‐induced rat model of Alzheimer's disease (Khorasani et al., 2019). Rafiepour and colleagues reported that PPARγ‐dependent signaling is involved in protective effects of ABA on 6‐OHDA‐induced SH‐SY5Y cell apoptosis (Rafiepour et al., 2019).

Previous studies have shown that the activation of PPARγ protects neurons against NMDA excitotoxicity currents (Pancani et al., 2009; Zhao et al., 2006). Surprisingly, it has been reported that the PPARγ agonist (pioglitazone) improves tremor scores, motor disturbances, and spatial learning and memory impairments (Ihm et al., 2010). Furthermore, it is possible that inhibition of glutaminergic currents prevent tremor‐induced impairments.

This study supported the ability of ABA to prevent tremor‐induced deficits. However, the administration of ABA alone is also necessary to endorse the role of ABA in improvement of these deficits.

In conclusion, the findings of this study confirmed that harmaline induces ET and motor disturbance as well as impairs passive avoidance learning. Central administration of ABA could ameliorate harmaline‐induced tremor in a rat model of ET. Abscisic acid could attenuate locomotor and cognitive impairments induced by harmaline. The exact mechanism of ABA in harmaline‐induced tremor needs to be evaluated in further experimental studies.

## CONFLICT OF INTEREST

The authors declare no conflict of interest.

### PEER REVIEW

The peer review history for this article is available at https://publons.com/publon/10.1002/brb3.2564

## Data Availability

The datasets used or analyzed during the current study are available from the corresponding author on reasonable request.
